# Supplementary material for: Contribution of Second Trimester Sonographic Placental Morphology to Uterine Artery Doppler in the Prediction of Placenta-Mediated Pregnancy Complications
Source: J Clin Med. 2022 Nov 15;11(22):6759. doi: 10.3390/jcm11226759 (PMC9697802; doi:10.3390/jcm11226759)
Supplement: Supplementary file 1 [file jcm-11-06759-s001.zip › jcm-1936760-supplementary.pdf]

Supplementary Material

Supplemental Table S1: Median values of placental sonographic markers by week

Gestational week	N	Length (cm)	Width (cm)	Thickness (cm)	Area (cm^2)	Volume (cm^3)	Mean uterine artery PI
16	43	11.72	10.22	2.40	120.48	304.42	1.30
17	15	13.02	11.35	2.57	147.10	369.68	1.21
18	18	13.13	11.75	2.60	156.04	404.38	1.14
19	67	14.00	12.47	2.57	177.06	446.35	1.07
20	42	15.17	13.22	2.63	198.15	539.73	0.97
21	70	15.17	13.22	2.63	198.15	539.73	0.97
22	96	15.37	13.38	2.77	206.55	566.80	1.00
23	47	16.03	14.05	2.93	231.51	628.05	0.98
24	31	16.50	14.25	3.17	238.89	679.37	0.98

PI, pulsatility index
Values are derived from the overall study cohort of 429 women who underwent sonographic placental study using the same protocol employed in the current study.
